# Scissor sisters: regulation of ADAM10 by the TspanC8 tetraspanins

**DOI:** 10.1042/BST20160290

**Published:** 2017-06-15

**Authors:** Alexandra L. Matthews, Justyna Szyroka, Richard Collier, Peter J. Noy, Michael G. Tomlinson

**Affiliations:** School of Biosciences, College of Life and Environmental Sciences, University of Birmingham, Edgbaston, Birmingham B15 2TT, U.K.

**Keywords:** ADAM10, metalloprotease, tetraspanin, TspanC8

## Abstract

A disintegrin and metalloprotease 10 (ADAM10) is a ubiquitously expressed transmembrane protein which is essential for embryonic development through activation of Notch proteins. ADAM10 regulates over 40 other transmembrane proteins and acts as a ‘molecular scissor’ by removing their extracellular regions. ADAM10 is also a receptor for α-toxin, a major virulence factor of *Staphylococcus aureus*. Owing to the importance of its substrates, ADAM10 is a potential therapeutic target for cancer, neurodegenerative diseases such as Alzheimer's and prion diseases, bacterial infection and inflammatory diseases such as heart attack, stroke and asthma. However, targetting ADAM10 is likely to result in toxic side effects. The tetraspanins are a superfamily of 33 four-transmembrane proteins in mammals which interact with and regulate specific partner proteins within membrane nanodomains. Tetraspanins appear to have a cone-shaped structure with a cholesterol-binding cavity, which may enable tetraspanins to undergo cholesterol-regulated conformational change. An emerging paradigm for tetraspanin function is the regulation of ADAM10 by the TspanC8 subgroup of tetraspanins, namely Tspan5, 10, 14, 15, 17 and 33. This review will describe how TspanC8s are required for ADAM10 trafficking from the endoplasmic reticulum and its enzymatic maturation. Moreover, different TspanC8s localise ADAM10 to different subcellular localisations and may cause ADAM10 to adopt distinct conformations and cleavage of distinct substrates. We propose that ADAM10 should now be regarded as six different scissor proteins depending on the interacting TspanC8. Therapeutic targetting of specific TspanC8/ADAM10 complexes could allow ADAM10 targetting in a cell type- or substrate-specific manner, to treat certain diseases while minimising toxicity.

## Introduction

Cell development and function are regulated by cell surface receptors and secreted proteins that co-ordinate intra- and intercellular signalling. Various regulatory mechanisms are required to ensure appropriate spatiotemporal control over these biological processes. Proteolytic cleavage, or ‘shedding’, of the ectodomains (extracellular regions) at a juxta-membrane site on transmembrane proteins has emerged as one such mechanism. Ectodomain shedding directly affects the responsiveness of cells to extracellular signals via activation of intracellular signalling or down-regulation of cell surface receptors, or indirectly through the release of soluble mediators from their membrane-bound precursors. Shedding therefore enables cells to rapidly respond to their environment and plays an important role in copious cellular processes including cell adhesion, migration, invasion, proliferation and signalling [1,2].

## ADAM10: a ubiquitous ‘molecular scissor’

**Figure 1.**
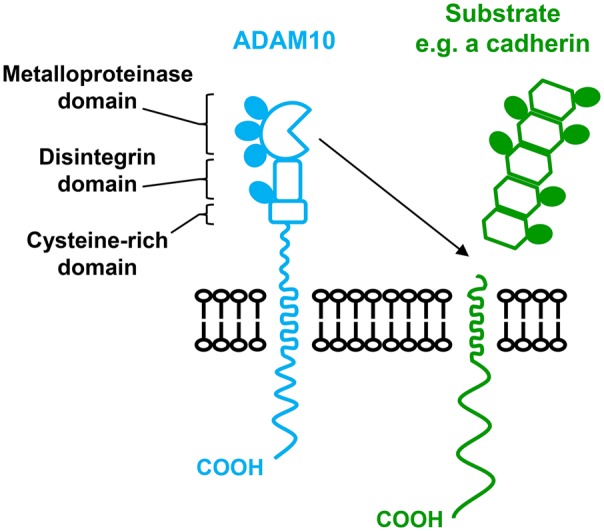
Diagrammatic representation of ADAM10 cleaving a substrate. N-glycosylation sites are indicated by filled ovals.

The evolutionarily conserved ADAMs (a disintegrin and metalloproteases) are a superfamily of Zn^2+^-dependent transmembrane metalloproteases that are responsible for a substantial proportion of transmembrane protein shedding. A total of 22 ADAM genes have been identified in humans, of which 12 encode proteolytically active enzymes. ADAMs share a common multidomain structure consisting of an N-terminal signal sequence, followed by a prodomain, a metalloprotease domain, a disintegrin domain, a cysteine-rich region, an epidermal growth factor (EGF) domain (except ADAM10 and ADAM17), a transmembrane region and a cytoplasmic tail (Figure 1) [3]. Metalloproteases are synthesised as zymogens and undergo maturation during biosynthesis. This is the process by which the prodomain, which acts as a chaperone and an inhibitor of enzyme activity, is cleaved by proprotein convertases [4].

ADAM10 is ubiquitously expressed in mammalian cells and is one of the best studied ADAMs with over 40 substrates identified [5,6]. ADAM10 is essential for embryonic development, because ADAM10-deficient mice die at embryonic day 9.5 and display a defective neuronal and vascular phenotype [7]. This is comparable with features observed in mice deficient for the cell fate regulator Notch1. Indeed, ADAM10 is responsible for the ligand-dependent cleavage of Notch proteins, which enables the membrane-tethered fragment of Notch to undergo intramembrane proteolysis within its transmembrane region by γ-secretase. This results in the translocation of the Notch intracellular domain to the nucleus where it functions as a transcriptional regulator [8–11].

A second well-studied ADAM10 substrate is the amyloid precursor protein (APP). Cleavage of the APP by β- and γ-secretases generates the pathogenic amyloid peptide Aβ, which forms amyloid plaques and has been associated with Alzheimer's disease. ADAM10 can prevent the formation of the Aβ peptide in a mouse model by cleaving APP at a site within the Aβ peptide region [12]. Thus, induction of ADAM10 activity has therapeutic potential for the treatment of Alzheimer's disease. Similarly, ADAM10 activation has been proposed as a potential treatment for prion disease, because ADAM10 sheds the cellular prion protein and conditional deletion of ADAM10 in forebrain neurones reduces survival time in a mouse model of prion disease [13]. However, the role of ADAM10 in prion disease is complicated, because ADAM10 also reduces spread of the disease throughout the brain and reduces neuropathological consequences [13]. Induction of ADAM10 activity is also a potential treatment for arterial thrombosis, which leads to heart attack and ischaemic stroke. ADAM10 sheds the platelet-activating collagen receptor glycoprotein VI (GPVI) [14,15], and GPVI deficiency protects against arterial thrombosis in mouse models [16]. In contrast with these therapeutic possibilities to activate ADAM10, inhibition of ADAM10 has potential for the treatment of other diseases. ADAM10 may promote cancer progression by shedding and release of EGF receptor ligands betacellulin and EGF [17], for example, and may promote asthma by cleaving the low-affinity immunoglobulin E receptor CD23 [18,19]. ADAM10 may also promote inflammatory diseases by releasing chemokines CX3CL1 and CXCL16 [20], and by weakening epithelial and endothelial cell–cell junctions by cleaving epithelial (E), neuronal (N) and vascular endothelial (VE) cadherins [21–23]. In addition, ADAM10 is a receptor for *Staphylococcus aureus* α-toxin, which activates ADAM10 to cleave cadherins on epithelial and endothelial cells. This disrupts barrier formation to promote tissue damage and spread of the bacteria [24]. Thus, ADAM10 has a wide range of substrates with important roles in health and disease, and ADAM10 shedding can be disease-preventing or disease-promoting, depending on the substrate.

The regulation of ADAM10 activity is not clear. ADAM10 appears to have constitutive activity towards some substrates, although its activity can be up-regulated by certain stimuli that induce intracellular signals, such as an increase in intracellular Ca^2+^ concentration. The mechanism underlying the latter is not well understood, but is dependent on the transmembrane region of ADAM10 rather than the cytoplasmic tail [25]. There is also evidence that ADAM10-mediated shedding may be facilitated by intracellular signals that modify the substrates, because phosphorylation of the cytoplasmic tail of the adhesion molecule CD44 may induce dimerisation and/or a conformational change in the CD44 ectodomain to allow shedding [26,27].

ADAM10 is clearly a promising therapeutic target for a range of diseases, but because of the positive and negative effects of ADAM10 in health and disease processes, substrate-specific ADAM10 targetting may be necessary to avoid toxic side effects. The remainder of this review will describe the emerging role of the TspanC8 tetraspanins as ADAM10-interacting partners that are essential for its exit from the endoplasmic reticulum, and which may dictate substrate specificity.

## Tetraspanins regulate partner protein function

The regulation of transmembrane proteins by compartmentalisation into membrane microdomains is a concept that has developed from studies of lipid rafts, caveolae, neuronal and immunological synapses, and tetraspanins. The latter are a superfamily of four-transmembrane proteins that are expressed by animals, plants and some multicellular fungi. They function by interacting with specific ‘partner proteins’, and regulating their intracellular trafficking and lateral mobility and clustering at the cell surface [28,29]. Notable examples are regulation of laminin-binding integrin function by tetraspanin CD151 [29], regulation of B-cell co-receptor CD19 trafficking by tetraspanin CD81 [30] and regulation of the Wnt receptor Frizzled-4 by Tspan12 [31]. In each case, tetraspanin mutations lead to human diseases that are consistent with functional impairment of the partner protein: CD151 deficiency leads to kidney disease due to impaired assembly of the glomerular and tubular basement membranes [32]; CD81 deficiency leads to impaired antibody generation due to an absence of CD19 on the B-cell surface [33]; and Tspan12 deficiency leads to familial exudative vitreoretinopathy characterised by impaired vasculature development in the retina [34,35]. A total of 33 tetraspanins are found in humans and each cell type is estimated to express as many as 20 different tetraspanins. Tetraspanins form dynamic nanoclusters that are distinct from lipid rafts [28,29], and recent evidence using super-resolution microscopy suggests that tetraspanin nanodomains are clusters of ∼10 tetraspanins of just a single type [36]. This is consistent with the specific phenotypes that have been observed in many tetraspanin knockout studies, although tetraspanin knockouts are typically well tolerated and there is evidence for functional compensation by related tetraspanins [28,29].

**Figure 2.**
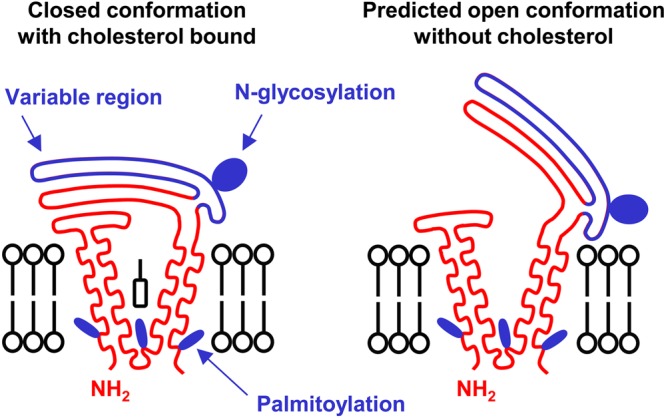
Diagrammatic representation of a tetraspanin in closed and open conformations. The variable region of the major extracellular region is indicated in blue. Tetraspanins have between 0 and 3 N-linked glycosylation sites and several cysteine residues which can be palmitoylated (indicated in blue). This model is based on the crystal structure of tetraspanin CD81 [37]; additional structures are required to determine whether this is a common structure for all tetraspanins.

An exciting breakthrough in tetraspanin research is the recent report of the first crystal structure of a full-length tetraspanin [37]. The Blacklow group showed that tetraspanin CD81 has a cone-like structure in which transmembranes 1 and 2 are separated at the top of the plasma membrane from transmembranes 3 and 4 (Figure 2A). This forms a cavity in which a cholesterol molecule can bind, with its hydroxyl group co-ordinated by conserved polar amino acids within transmembranes 1 and 4. Molecular dynamics simulations suggest that removal of the cholesterol might result in a dramatic conformational change from a ‘closed’ to an ‘open’ conformation, in which the main extracellular region swings upwards with a ‘switch-blade’-type action (Figure 2B) [37]. Structural determination of additional tetraspanins is now required to discover whether this structure is common to the entire superfamily. Nevertheless, this raises the possibility of therapeutic targetting with small molecules or antibodies that may lock the tetraspanin into a particular conformation. Hypothetically, this could then affect partner protein function, for example, by disrupting the interaction with the tetraspanin, by affecting localisation of the complex (e.g. causing internalisation) or by preventing conformational change-induced regulation by the tetraspanin.

## The emergence of the six TspanC8 tetraspanins as ADAM10 regulators

**Figure 3.**
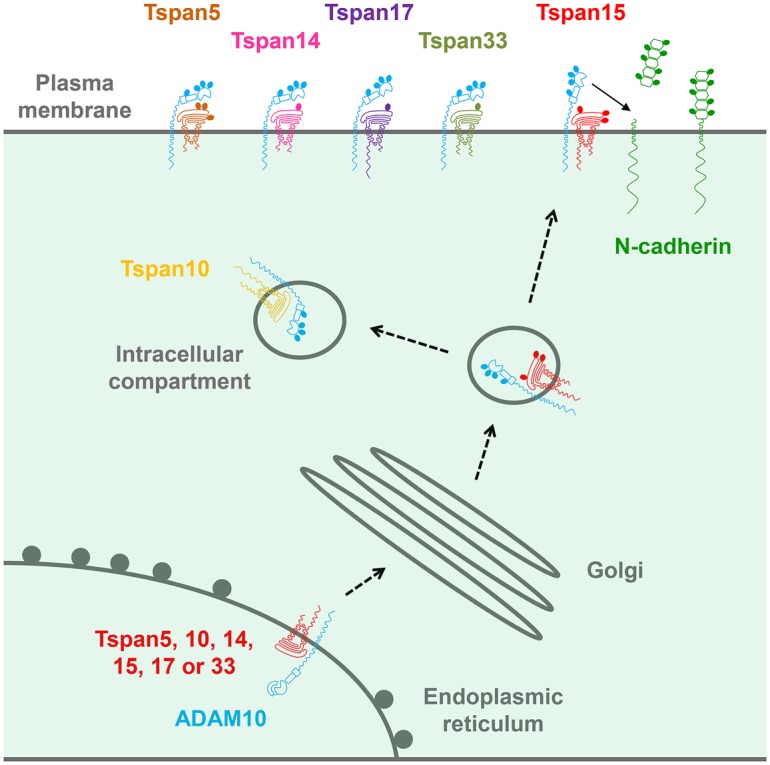
The six TspanC8s regulate ADAM10 trafficking and maturation. TspanC8s interact with ADAM10 and promote its trafficking out of the endoplasmic reticulum and its maturation, during which the inhibitory prodomain is cleaved. TspanC8s may traffic ADAM10 to the cell surface or to intracellular compartments (e.g. Tspan10 appears to be largely intracellular). TspanC8s promote ADAM10 specificity for certain substrates (e.g. Tspan15 promotes N-cadherin cleavage).

ADAM10 is one of the most commonly identified tetraspanin-associated proteins in proteomic studies, and the majority of ADAM10 appears to be tetraspanin-associated [38]. In 2012, we and others reported this to be mediated by an interaction with any one of six largely unstudied tetraspanins that are particularly related by protein sequence [39–41]. These are Tspan5, 10, 14, 15, 17 and 33, which we termed the TspanC8 subgroup because they are unique among tetraspanins in possessing eight cysteines within their main extracellular region [39,40]. TspanC8s promote ADAM10 exit from the endoplasmic reticulum and removal of its inhibitory prodomain, and most TspanC8s promote trafficking to the cell surface (Figure 3) [39–41]. This mechanism holds true in *Drosophila* [39], primary human endothelial cells [40] and in red blood cells through our study of Tspan33 knockout mice [40], so far the only reported TspanC8 knockout mouse [42], together indicating that TspanC8s are fundamental to ADAM10 function.

**Figure 4.**
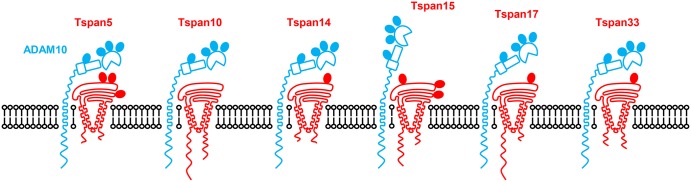
Diagrammatic representation of the six different TspanC8/ADAM10 complexes. The different conformations of ADAM10 in complex with different TspanC8s are hypothetical, but are based on interaction mapping data by Noy et al. [45]. N-glycosylation sites are indicated by filled ovals, but palmitoylation sites are not shown.

Several follow-up studies on TspanC8 regulation of ADAM10 [43–46] can be condensed into the following key findings: (1) different cell types have distinct repertoires of TspanC8 expression; (2) different TspanC8s have distinct subcellular localisations and lateral diffusion; (3) different TspanC8s interact with distinct regions of the ADAM10 extracellular region, suggesting that ADAM10 adopts different conformations in complex with different TspanC8s and (4) different TspanC8s can promote, or inhibit, ADAM10 cleavage of distinct substrates. These findings, which will be expanded on in the rest of this review, have led us to propose that ADAM10 can no longer be regarded as one molecular scissor. Instead, ADAM10 exists as six different scissors with different substrate specificities, depending on which TspanC8 it is associated with (Figure 4) [44].

It is worth noting that prior to the studies on TspanC8s, the non-TspanC8 tetraspanin Tspan12 was implicated in ADAM10 regulation [47]. However, this is now thought to be an indirect effect, because unlike TspanC8s, no Tspan12 co-immunoprecipitation with ADAM10 is detected under stringent lysis conditions [39,40]. More recently, Tspan3 has been shown to interact with ADAM10 and APP [48]. Tspan3 is not a TspanC8, but forms a subgroup of inter-related tetraspanins that include Tspan6 and 7. Unlike TspanC8s, Tspan3 overexpression does not promote ADAM10 maturation or trafficking to the cell surface, but does promote APP cleavage. No effects on APP cleavage are detectable in brains of Tspan3 knockout mice, but this could be due to compensation by the related Tspan7, which is up-regulated at the mRNA level in the absence of Tspan3 [48]. Additional studies are needed to determine how ADAM10 is regulated by Tspan3, and possibly by Tspan6 and 7, and their potential interplay with TspanC8s.

## Different cell types have distinct TspanC8 repertoires

**Figure 5.**
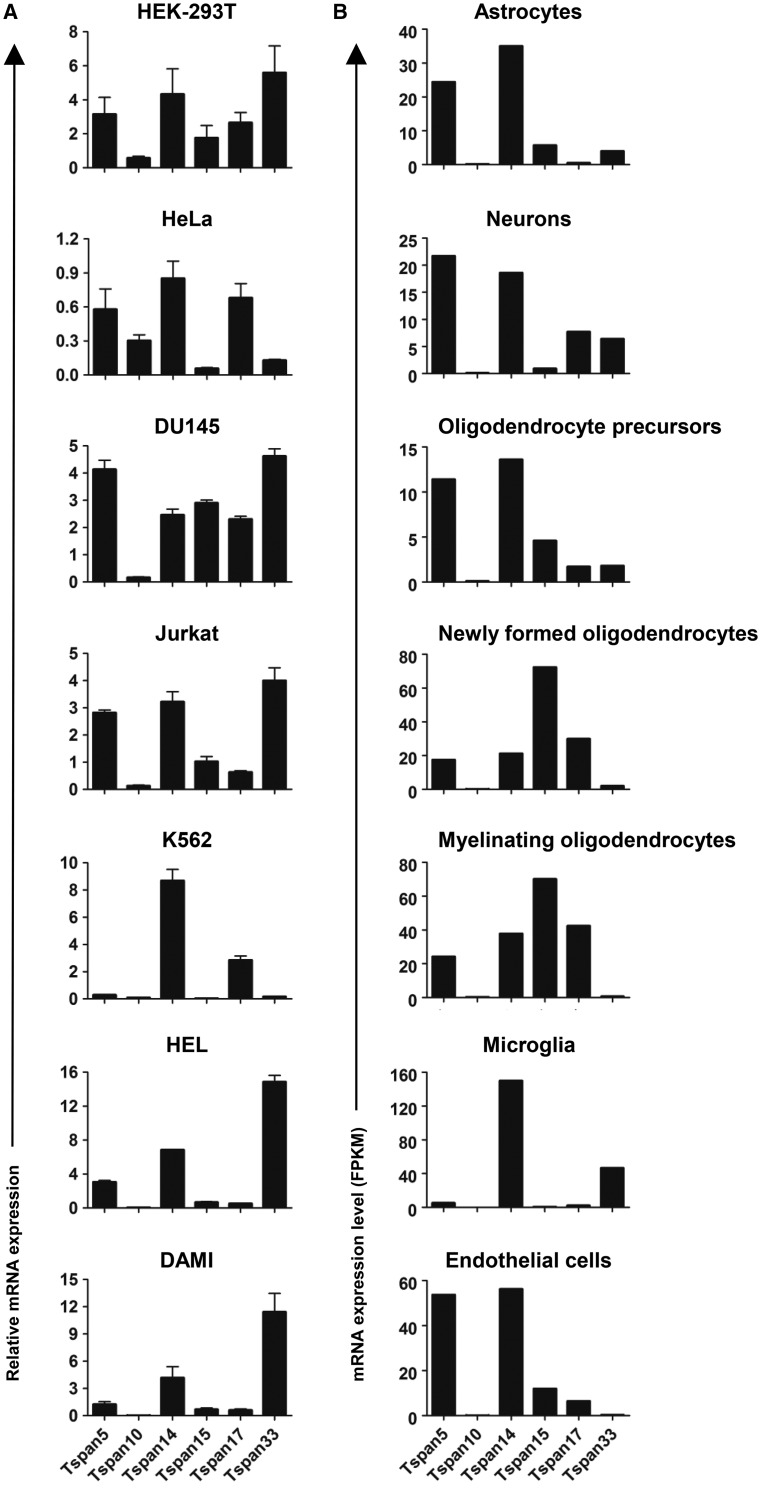
Different cell types express distinct repertoires of TspanC8s. (**A**) The mRNA levels of TspanC8s in different human cell lines were measured by quantitative real-time polymerase chain reaction, as previously described [40]. The cell lines were human embryonic kidney 293 cells expressing the large T antigen of SV40 virus (HEK-293T), HeLa epithelial cells, DU145 prostate cancer cells, Jurkat T cells, K562 chronic myelogenous leukaemia cells, human erythroleukaemia cells (HEL) and DAMI megakaryoblast cells. (**B**) An RNA-Seq dataset from mouse brain for seven major cell types, generated by Zhang et al. [49], was used to present mRNA expression levels for TspanC8s as fragments per kilobase of transcript sequence per million mapped fragments (FPKM).

In the absence of effective antibodies to most TspanC8s, we have used RT-PCR to determine mRNA expression levels of TspanC8s in different cell types. Primary human umbilical vein endothelial cells and the A549 lung epithelial cell line express most of the TspanC8s, but primary mouse erythroblasts and megakaryocytes express predominantly one TspanC8, namely Tspan33 and Tspan14, respectively. Analyses of an additional eight human cell lines suggest that expression of most of the six TspanC8s is a common feature, albeit with considerable variation in relative levels between cell types (Figure 5A). Similarly, examination of TspanC8 expression within an RNA-Seq dataset, from seven major cell types in mouse brain [49], indicates that most cell types express most TspanC8s, but at levels specific to the cell type (Figure 5B). Oligodendrocyte precursors express five TspanC8s, with highest expression of Tspan5 and Tspan14. This pattern is similar in astrocytes and neurones, but in myelinating oligodendrocytes Tspan15 is the most highly expressed and Tspan33 is lost. The macrophages of the brain, the microglia, express Tspan14 and Tspan33, while brain endothelial cells express Tspan5, 14, 15 and 17 (Figure 5B) [49], similar to human umbilical vein endothelial cells [40]. Given that ADAM10 has several substrates in the brain, including APP and cellular prion protein [6], it will be of interest to investigate brain function in TspanC8 knockout mice as they become available. Taken together, it is clear that different cell types have different repertoires of ADAM10 scissors, providing ample scope for differential substrate shedding and the potential for therapeutic targetting in a cell type- or substrate-specific manner by targetting specific TspanC8/ADAM10 complexes.

## Different TspanC8s have distinct subcellular localisations and lateral diffusion

The Rubinstein group have investigated TspanC8 subcellular localisation by transfecting green fluorescent protein (GFP)-tagged TspanC8s into human cell lines. Tspan5, 14, 15 and 33 promote ADAM10 surface localisation in the HeLa epithelial cell line, but Tspan10 and 17 do not. This is consistent with substantial localisation of the latter in an intracellular compartment, but cell surface and intracellular localisation of the other four TspanC8s [39]. In subsequent comparisons between GFP-tagged Tspan5 and 15 expressed in the U2OS osteosarcoma cell line, ADAM10 lateral diffusion in the plasma membrane is increased by 55% in the presence of Tspan15, but Tspan5 has no effect [43]. In addition, co-immunoprecipitation followed by mass spectrometry-based proteomics identifies many common interacting proteins, most prominently ADAM10, but some proteins are more specific to Tspan5 or Tspan15. Additionally, overexpression of Tspan5 or Tspan15 appears to alter the proteins interacting with ADAM10 [43]. These data suggest that TspanC8s differentially regulate ADAM10 localisation, but interpretation is complicated by the fact that the GFP tag and/or overexpression might affect localisation. TspanC8 monoclonal antibodies are now required to investigate the localisation of endogenous proteins.

## Different TspanC8s may cause ADAM10 to adopt distinct conformations

We have demonstrated that the main extracellular region of Tspan14 mediates the interaction with ADAM10 and its maturation, and that the variable region is necessary but not sufficient [45]. This study used co-immunoprecipitation of overexpressed FLAG epitope-tagged constructs chimeric for Tspan14 and the non-TspanC8 tetraspanin CD9. A similar strategy with ADAM10 truncation constructs and chimeras with ADAM17, which does not interact with TspanC8s, showed some interesting differences between TspanC8s. Tspan15 requires only the membrane–proximal stalk region of ADAM10 for its interaction, Tspan17 requires the stalk and cysteine-rich domain, whereas Tspan5, 10, 14 and 33 require the stalk, cysteine-rich and disintegrin domains [45]. These findings suggest that ADAM10 might adopt different conformations in complex with different TspanC8s, as suggested in Figure 4. However, interpretation of these data is complicated by their reliance on co-immunoprecipitation of overexpressed mutant constructs, and thus, high-resolution structural studies of native TspanC8/ADAM10 complexes are now required.

The cleavage sites on ADAM10 substrates share no particular amino acid sequence similarities and differ in their position relative to the plasma membrane. For example, the GPVI, APP and Notch1 cleavage sites are positioned at 5, 12 and 15 amino acids from the transmembranes, respectively [11,15,50]. Therefore, if different TspanC8s are able to ‘lock’ ADAM10 into a distinct conformation, this could position the ADAM10 metalloprotease domain at a position that favours cleavage of certain substrates.

## Different TspanC8/ADAM10 complexes cleave distinct substrates

The most definitive evidence for cleavage of specific ADAM10 substrates by different TspanC8/ADAM10 complexes is the promotion of N-cadherin cleavage by Tspan15/ADAM10. This is because the experiments have compared Tspan15 with other TspanC8s as controls have been reported by three different groups using different cell lines and the effects are striking. Indeed, Tspan15 overexpression substantially promotes N-cadherin cleavage, but other TspanC8s do not [41,43,45]. Furthermore, Tspan15 knockdown substantially reduces N-cadherin cleavage [43].

Notch cleavage is also regulated by TspanC8s [39,43,46,51], but interpretation of these findings is complicated by the positive and negative effects described for different TspanC8s, the different cell types used and the different methods of activating Notch and detecting its cleavage. In the two studies which have compared different TspanC8s, ligand-induced Notch reporter activity in HeLa and U2OS cells is shown to be promoted by overexpression of Tspan5 or 14, whereas Tspan15 or 33 has the opposite effect. Moreover, Tspan15 knockdown can substantially promote Notch activation [39,43]. In additional studies which focused on just one or two TspanC8s, it was reported that knockdown of Tspan5 or 10 in primary mouse osteoclasts inhibits Notch cleavage and target gene expression [46], whereas Tspan33 has a positive role in expression of Notch target genes in the RAW 264.7 mouse macrophage cell line [51]. Thus, Tspan5, 10 and 14 appear to be Notch-promoting, Tspan15 Notch-inhibiting and Tspan33-dependent on the cell type. A panel of CRISPR/Cas9-generated cell lines, expressing single TspanC8s, may ultimately be required to understand the importance of each TspanC8 in the activation of Notch and potentially other ADAM10 substrates.

Recent additional findings from cell line models suggest that GPVI, CD44 and APP cleavage may also be regulated by specific TspanC8s. GPVI cleavage is inhibited by overexpression of Tspan14 but not by other TspanC8s [45], and CD44 cleavage is partially dependent on Tspan5 but not on Tspan15 [43]. Finally, regulation of APP cleavage by TspanC8s is less clear, because both positive [41] and negative [43] effects of Tspan15 have been reported. This may reflect the different cell lines used and their potentially different endogenous TspanC8 repertoires.

Finally, genetic screens in cell lines have recently shown Tspan14 and Tspan33 to be important for cytotoxicity induced by *S. aureus* α-toxin; the screens also identified the α-toxin receptor ADAM10 [52,53]. One explanation for their identification would be that these TspanC8s are highly expressed in the cell lines screened and are required for ADAM10 surface expression, which certainly appears to be the case for Tspan14 in the U937 human monocyte cell line [53]. However, it is possible that TspanC8s might differentially regulate ADAM10 function as an α-toxin receptor.

The mechanisms by which TspanC8s differentially control ADAM10 substrate specificity are unclear, but the regulation of ADAM10 subcellular localisation and conformation, proposed in the previous sections, represent potential mechanisms. An additional possibility is direct interaction of the TspanC8 with a particular substrate. However, although at least two ADAM10 substrates are tetraspanin-associated, namely CD44 [54] and GPVI [55,56], there is no current evidence that a TspanC8 can interact with these proteins.

## TspanC8 protein sequence analyses: conserved transmembrane and extracellular regions; divergent tails

**Figure 6.**
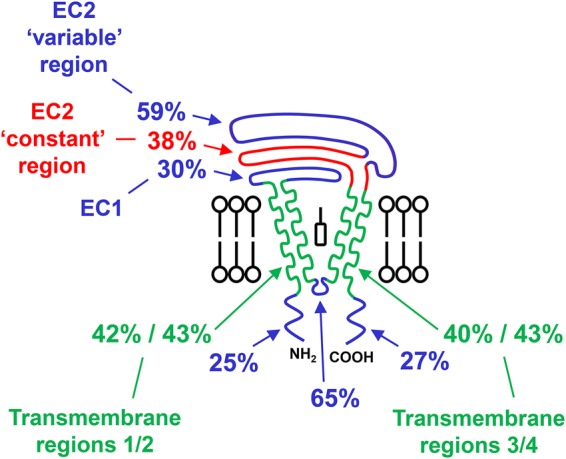
The variable region of the main extracellular domain is the most highly conserved region of TspanC8s, and the cytoplasmic tails are the least conserved. Amino acid sequence comparisons for each region of the six human TspanC8s were performed using the Clustal Omega multiple sequence alignment tool [57]. The average sequence identities were calculated for each region. Note that the cytoplasmic tails are even more divergent than indicated, because the sequence alignment does not factor in the different tail lengths (as indicated in Figure 4). EC1 refers to the small extracellular region and EC2 refers to the main extracellular region.

In the current absence of useful tools such as TspanC8 monoclonal antibodies and knockout mice (apart from Tspan33 [40,42]), comparisons of TspanC8 protein sequences may help us to understand how they differentially regulate ADAM10. TspanC8s are certainly relatively highly related by amino acid sequence [40], with the human homologues ranging from 78% amino acid identity between Tspan5 and 17 to 26% identity between the most distantly related TspanC8s, as determined by Clustal Omega multiple sequence alignment [57] (Table 1). The large extracellular region is relatively highly conserved between TspanC8s (average 44% sequence identity), with the ‘variable’ region even more conserved (59%) (Figure 6). This contrasts strikingly with sequence comparisons within the tetraspanins as a whole, in which the variable region varies most between tetraspanins. However, this is consistent with our finding that the interaction with ADAM10 is mediated by the main extracellular region of the TspanC8s and that the variable region is essential [45], suggesting that this ADAM10-interacting region has been conserved during evolution. The transmembrane domains of TspanC8s are also well conserved (40–43%), consistent with their forming the core tetraspanin structure [37].

**Table 1:** Amino acid sequence identities for the human TspanC8s Sequence analyses were performed using the Clustal Omega multiple sequence alignment tool [57] and presented as percentage identity.

	Tspan10	Tspan33	Tspan14	Tspan5	Tspan17
Tspan15	26.3	34.0	31.6	32.6	30.2
Tspan10		32.1	32.5	34.5	33.9
Tspan33			40.1	42.3	42.7
Tspan14				57.8	57.0
Tspan5					78.4

The cytoplasmic tails of TspanC8s are relatively divergent. The 25% and 27% sequence identities of the N- and C-terminal tails, respectively (Figure 6), actually under-represent the true diversity, because the sequence alignment tool does not factor in the substantially different lengths of the TspanC8 tails (Figure 4). These are unusually long in TspanC8s, averaging 29 and 36 amino acids for the N- and C-tail, respectively, and as long as 77 amino acids for the Tspan10 N-terminus and 74 amino acids for the Tspan17 C-terminus. This compares with 10–15 amino acids for most other tetraspanins. The functions of the cytoplasmic regions of TspanC8s are not known, although Tspan10 (previously named oculospanin) and Tspan15 (previously named NET-7) have putative dileucine-based and tyrosine-based intracellular sorting motifs, respectively [58]. Thus, cytoplasmic regions could engage with intracellular trafficking proteins and be partly responsible for the distinct TspanC8 subcellular localisations which have been reported [39,43]. As a precedent for such a mechanism, the C-terminus of tetraspanin CD63 binds to the PDZ domain-containing protein syntenin-1, which regulates the constitutive internalisation of the tetraspanin [59]. ADAM10 trafficking to neuronal synapses is regulated by interaction with the PDZ domain-containing synapse-associated protein-97 (SAP97) [60], but it is unclear whether TspanC8s could also interact with SAP97. ADAM10 trafficking to the cell surface is also regulated by Rab14a and its guanine nucleotide exchange factor FAM116A [61], but no direct association between ADAM10 and Rab14a/FAM116a has been reported. Thus, it is possible that the cytoplasmic tails of TspanC8 tetraspanins may play a role in the trafficking of ADAM10, by interacting with Rab GTPases and/or their effectors.

## Future directions

Cell biology experiments will aim to determine the molecular mechanisms by which TspanC8s localise ADAM10 to distinct substrates. Biophysical methods will investigate whether ADAM10 adopts different conformations in complex with different TspanC8s. It will also be important to generate antibodies and small molecules to modulate specific TspanC8/ADAM10 complexes with the ultimate goal of disease treatment. Finally, since it is well established that cholesterol depletion activates ADAM10 [62–64], could this be due to a conformational change in TspanC8s, which serves as a trigger for ADAM10 activation?

## Abbreviations

ADAM: a disintegrin and metalloprotease
APP: amyloid precursor protein
EGF: epidermal growth factor
GFP: green fluorescent protein
GPVI: glycoprotein VI
SAP97: synapse-associated protein-97
